# Does dietary calcium interact with dietary fiber against colorectal cancer? A case–control study in Central Europe

**DOI:** 10.1186/1475-2891-12-134

**Published:** 2013-10-04

**Authors:** Aleksander Galas, Malgorzata Augustyniak, Elzbieta Sochacka-Tatara

**Affiliations:** 1 Department of Epidemiology, Chair of Epidemiology and Preventive Medicine, Jagiellonian University – Medical College, Kopernika St 7a, Krakow 31-034, Poland

**Keywords:** Colorectal cancer, Diet, Calcium, Fiber, Effect modification, Case–control study

## Abstract

**Background:**

An unfavorable trend of increasing rates of colorectal cancer has been observed across modern societies. In general, dietary factors are understood to be responsible for up to 70% of the disease’s incidence, though there are still many inconsistencies regarding the impact of specific dietary items. Among the dietary minerals, calcium intake may play a crucial role in the prevention. The purpose of this study was to assess the effect of intake of higher levels of dietary calcium on the risk of developing of colorectal cancer, and to evaluate dose dependent effect and to investigate possible effect modification.

**Methods:**

A hospital based case–control study of 1556 patients (703 histologically confirmed colon and rectal incident cases and 853 hospital-based controls) was performed between 2000–2012 in Krakow, Poland. The 148-item semi-quantitative Food Frequency Questionnaire to assess dietary habits and level of nutrients intake was used. Data regarding possible covariates was also collected.

**Results:**

After adjustment for age, gender, education, consumption of fruits, raw and cooked vegetables, fish, and alcohol, as well as for intake of fiber, vitamin C, dietary iron, lifetime recreational physical activity, BMI, smoking status, and taking mineral supplements, an increase in the consumption of calcium was associated with the decrease of colon cancer risk (OR = 0.93, 95% CI: 0.89-0.98 for every 100 mg Ca/day increase). Subjects consumed >1000 mg/day showed 46% decrease of colon cancer risk (OR = 0.54, 95% CI: 0.35-0.83). The effect of dietary calcium was modified by dietary fiber (p for interaction =0.015). Finally, consistent decrease of colon cancer risk was observed across increasing levels of dietary calcium and fiber intake. These relationships were not proved for rectal cancer.

**Conclusions:**

The study confirmed the effect of high doses of dietary calcium against the risk of colon cancer development. This relationship was observed across different levels of dietary fiber, and the beneficial effect of dietary calcium depended on the level of dietary fiber suggesting modification effect of calcium and fiber. Further efforts are needed to confirm this association, and also across higher levels of dietary fiber intake.

## Background

Despite the many public health interventions to prevent the development of cancer - including colorectal cancer - that have been undertaken during the last decades, increasing prevalence of colorectal cancer across modern societies is still observed. According to the WHO, colorectal cancer amounts to approximately 1 234 000 new cases annually, and accounts for 608 000 deaths worldwide [1]. There is a large available body of published scholarship on the role of diet in the development of this disease, though it presents with numerous inconsistencies. Nevertheless, it is accepted that dietary factors are responsible for up to 70% of colorectal cancer cases [2].

Calcium is a mineral that has been intensively investigated in terms of its role in the prevention of colorectal cancer. There are a fair amount of plausible biological mechanisms (indirect and direct) explaining the physiological role of calcium. The indirect pathway is expected to be the effect of the creation of insoluble bile acid complexes [3], thus decreasing the proliferative role of secondary bile acids [4]. The direct pathway is associated with the role of calcium played at the cellular level, and this pathway is also related to the action of vitamin D, as the effect of the intracellular concentration of calcium, activation of calmodulin, phosphorylation of some cellular enzymes and activation of signaling pathways leading to cell differentiation or apoptosis [5].

Despite the presence of a relatively good body of evidence from basic research (meaning an experimental or theoretical work undertaken primarily to acquire new knowledge of the underlying foundations of phenomena and observable facts, without any particular application or use in view [6]), human studies haven’t shown consistent results. Majority studies performed before the year 2000 failed to confirm the presence of a clear beneficial effect of calcium [7-10]. There have also been studies on the use of calcium supplements, showing an up to 34% reduction in a rate of colorectal adenoma recurrence among patients with a history of adenomas [11-13]. The results of most recent studies are also inconsistent [14,15]. However, there is increasing evidence suggesting that calcium plays a prophylactic role [16-18].

There are some possible explanations for the lack of an observed benefit from calcium on the development of colorectal cancer. One is the effect of dose, as some of the investigations evaluated the risk across relatively low dosages [19]; some others were not designed for that purpose [14]; some had relatively small sample sizes [12,20,21]; and finally, calcium - especially dietary calcium is one of several dietary components, and analyses performed probably did not enable researchers to isolate the effects of calcium from the role of some other dietary nutrients.

The purpose of this study was to assess the role of intake of higher levels (over 1000 mg/day) of dietary calcium on the risk of colorectal cancer, to evaluate dose dependent effect. Besides, there is evidence in colon cancer research showing protective effect of fiber [18,22] which is consumed in different food than calcium. This has led us to the hypothesis that intake of fiber may modify the effect of calcium.

## Methods

### Sample

This paper provides an analysis of three consecutive stages of a hospital-based case control study carried out between 2000 and 2012 in Krakow. Cases chosen for this study were of patients diagnosed and treated at the I Chair of General Surgery and Department of Gastroenterological Surgery, Jagiellonian University Medical College, Krakow, Poland and controls were adults admitted to the I Department of Internal Medicine Jagiellonian University Medical College and to the II Internal Ward at the Narutowicz Municipal Hospital in Krakow. The design of the study has been described elsewhere [23,24]. In brief, study participants were incident cases of colorectal cancer. All included cases consisted of histologically confirmed sporadic adenocarcinomas of either the colon or rectum. Control group patients were recruited at the same time from inpatients hospitalized due to other health problems. Exclusion criteria consisted of the following: age over 75, the presence of communication (verbal contact) problems and/or cognitive limitations, and diagnosis of any of the following: hereditary non-polyposis colorectal cancer, familiar adenomatous polyposis, attenuated familial adenomatous polyposis, mixed polyposis syndrome, Ashkenazi colon cancer, hereditary breast and colorectal cancer or any of hamartomatous polyposis syndrome, moreover, the diagnosis of secondary cancer (distant metastasis), the diagnosis of primary cancer other than colorectal, recurrent cancer, underwent surgery (before recruitment) of gastrointestinal tract, present or past diagnosis of chronic disease of gastrointestinal tract (diverticulitis, irritable bowel syndrome, acute or chronic gastric ulcer, acute/chronic pancreatitis), diabetes (any type), renal failure, hepatic insufficiency or the presence of prolonged gastrointestinal symptoms.

### Tools and data collection

Study participants were recruited at the time of their hospital admission. After written consent had been obtained, data was collected by trained interviewers during a face-to-face interview. A standardized questionnaire included questions about socio-demographic characteristic, dietary habits, alcohol consumption, smoking, physical activity and some other health behaviors. The design of the study was approved by the Bioethical Committee of Jagiellonian University, Krakow, Poland.

Dietary habits were evaluated by administration of a semi-quantitative food frequency questionnaire (SFFQ). The SFFQ was designed and developed with the cooperation with the German Cancer Research Centre while the tool development part of the EPIC-project (European Prospective Investigation into Cancer and Nutrition) was performed. In total, 148-dietary items were used in the SFFQ including questions about consumption of cereals, dairy products, bread, type and cuts of meat and fish (including preparation technique), fresh fruits (summer/winter time), salads and fresh and cooked vegetables, rice or pasta, soups, sweets, baked goods, drinks and others. For each food or beverage item, a commonly consumed portion size was specified by standardized photographs. Next, respondents were asked to provide information about frequency of consumption. For the research information about usual (habitual) consumption over the period of 1 year by calendar seasons was gathered by trained interviewers. Patient-cases were asked about their dietary patterns at a time of 5 years prior to the onset of gastrointestinal symptoms (if were present) or prior to the beginning of the diagnosis process. Control patients were asked about their usual diet 5 years before the interview. The Polish food composition tables were used to calculate the intake of macro and micronutrients [25,26]. The validity and reproducibility of the questionnaire was assessed and published elsewhere [27,28].

### Covariates

The protocol enabled collection of information about possible covariates. Physical activity from recreational activities was assessed over adult lifetime starting from age 20 through 30, 40, 50 and 60 years of age. Participants reported the average weekly time spent on 5 groups of activities: walking or hiking, bicycling, gardening, practicing sports and household activities, separately for summer and winter times. Additionally, they were asked about the amount of time spent on recreational activities which required at least moderate effort. Finally, the reported time spent on each activity was multiplied by its typical energy expenditure requirements according to 2011 Compendium of Physical Activities [29]. Because respondents reported total time spent on activities and didn’t consider brakes and a time within which they were able to reach the required level of effort, it was considered that 70% of reported time was spent effectively and these results were finally presented and used in the analyses. There were also some other covariates used, such as age, education, number of portions of fruits and raw and cooked vegetables consumed daily and of fish consumption weekly, alcohol consumption, vitamin C, Body Mass Index (in kg/m^2^), smoking (current, ex-smoker, non-smoker), taking mineral supplements and dietary iron (as a proxy measure of meat consumption).

### Statistical analysis

Groups were characterized by providing statistical mean and standard deviations as well as median with inter quartile range. The distributions of continuous variables were skewed (as tested by the Shapiro-Wilk test). To estimate statistical significance for the difference between groups the Mann–Whitney test was used. For categorical variables, significance was assessed by the chi-square test. The relative risk of the development of colorectal cancer as well as for colon and rectal cancers related to the different levels of dietary item intake was estimated by the odds ratios calculated by multivariable logistic regression models. Three major models were analyzed. One was used to estimate the effect of linear increase in consumption of calcium by 100 mg, the second to assess linear increase by 1000 mg and the third investigated the effect by categories meaning that the group of individuals consumed over 1000 mg dietary calcium per day was compared with the group who consumed less. Moreover, we performed analyzes considering three main logistic regression models (unadjusted; adjusted for demographic variables: age, gender, education; and fully adjusted model: adjusted for age, gender, education [primary school or less, vocational school, secondary/high school, university or higher], fish consumption [servings/week], fruit, raw and cooked vegetable consumption [servings/day], fiber [g/day], vitamin C [mg/day], dietary iron [<=14,03 mg/day; >14,03 mg/day], alcohol consumption [g/week], adult lifetime recreational physical activity [METs-h/week], BMI [kg/m^2^], smoking [current, ex-smokers, non-smokers], taking mineral supplements [yes/no]) for the whole group of colorectal cancer cases, as well as separately for colon and rectal cancers. Subsequently, an effect modification by calcium intake in the relationship between fiber consumption and colorectal cancer was investigated by testing an interaction term. Finally, sub-group differences considering the presence of interaction were analyzed. The *effmod* procedure (Stata Corp software) was used for this purpose. The *effmod* calculates user specified stratum specific odds ratios and confidence intervals for logistic regression models which include interaction terms between an exposure and effect modifiers. For missing data the pairwise deletion procedure was used. The statistical software package Stata/IC 11.2 for Windows, Stata Corp LP was used. The 'p’ value below 0.05 was considered statistically significant.

## Results

During the period 2000–2012, 703 colorectal cancer cases and 853 controls were recruited for the study. There were 341 (48.5%) colon and 362 (51.5%) rectal adenocarcinomas. Among controls, there were disorders of thyroid gland (E00-E07: 3.8%, mainly E04 'other nontoxic goiter’), hypertensive diseases (I10-I15: 11.7%, mainly I10 'primary hypertension’), ischemic heart disease (I20-I24: 19.7%, mainly I20 'angina pectoris’), other forms of heart disease (I30-I52: 3.9%, mainly I48 'atrial fibrillation and flutter’), diseases of respiratory system (J00-J99: 7.7%, mainly J18 'pneumonia’), hernia (K40-K46: 11.3%, mainly K40 'inguinal hernia’), disorders of gallbladder (K80-K87: 21.6%, mainly K80 'cholelithiasis’), syncope and collapse (R55: 2.1%), injuries (S00-S99: 4.7%, mainly S80-S89 'injuries to knee and lower leg’) and others (13.7%).

Basic characteristic of the study sample was presented in Table 1. Among colorectal cancer cases as compared to controls, there were more males (56.6% vs. 51.5%), cases were older (57.9 vs. 54.8 yrs) and there were less ex-smokers (25.5% vs. 31.3%) and more non-smokers (30.6% vs. 24.2%).

**Table 1:** Basic characteristic of study participants

	**Colorectal cancer cases**	**Controls**	**p**
	**[n = 703]**	**[n = 853]**	
Gender [n, (%)]			
Males	398 (56.6%)	439 (51.5%)	df = 1
Females	305 (43.4%)	414 (48.5%)	p^chi^ = 0.043
Age			
Mean, (SD)	57.9 (9.1)	54.8 (11.5)	
Median (Q1-Q3)	59 (52–65)	56 (47–64)	p^MW^ < 0.001
Education [n, (%)]	n = 701	n = 852	
Primary school or less	106 (15.1%)	126 (14.8%)	df = 3
Vocational school	185 (26.4%)	236 (27.7%)	p^chi^ = 0.947
Secondary/high school	250 (35.7%)	296 (34.7%)	
University or higher	160 (22.8%)	194 (22.8%)	
Recreational physical activity* [METs-h/week]			
Mean, (SD)	17.3 (16.0)	17.0 (15.4)	p^MW^ = 0.968
Median (Q1-Q3)	12.9 (5.6-24.5)	13.0 (5.8-24.4)	
BMI/body mass index [kg/m^2^]	n = 702	n = 846	
Mean, (SD)	27.3 (4.1)	27.2 (4.6)	p^MW^ = 0.411
Median (Q1-Q3)	26.9 (24.4-29.7)	26.7 (24.1-29.8)	
Smoking		n = 852	
Current smoker	309 (43.9%)	379 (44.5%)	df = 2
Ex-smoker	179 (25.5%)	267 (31.3%)	p^chi^ = 0.005
Non-smoker	215 (30.6%)	206 (24.2%)	

*chi*-chi-square test; *df* degrees of freedom, *MW* Mann–Whitney test.

* -adult lifetime recreational physical activity, i.e. the average for the period 18-current age (if current age <66) or for the period 18–65 (if current age >65).

Regarding foods and dietary items, we analyzed only those, which were considered as potential covariates. It was observed that colorectal cancer patients consumed significantly fewer servings of fish, alcohol, dietary calcium and dietary iron (Table 2).

**Table 2:** Intake of main dietary items

	**Colorectal cancer cases**	**Controls**	**p**
	**[n = 703]**	**[n = 853]**	
Fish consumption [servings/week]			
Mean (SD)	1.2 (1.2)	1.4 (1.4)	p^MW^ = 0.040
Median (Q1-Q3)	0.9 (0.5-1.5)	1.1 (0.6-1.8)	
Fruits [servings/day]			
Mean (SD)	1.8 (1.6)	2.3 (3.1)	
Median (Q1-Q3)	1.3 (0.7-2.5)	1.4 (0.7-3.0)	p^MW^ = 0.340
Raw vegetables [servings/day]			
Mean (SD)	1.2 (0.8)	1.3 (1.3)	
Median (Q1-Q3)	1.0 (0.6-1.5)	0.9 (0.6-1.6)	p^MW^ = 0.932
Cooked vegetables [servings/day]			
Mean (SD)	0.5 (0.3)	0.6 (0.3)	
Median (Q1-Q3)	0.5 (0.3-0.7)	0.5 (0.4-0.7)	p^MW^ = 0.135
Total energy [kcal/day]			
Mean (SD)	2236 (771)	2407 (1035)	
Median (Q1-Q3)	2088 (1725–2558)	2152 (1669–2857)	p^MW^ = 0.090
Pure alcohol [g/week]			
Mean (SD)	33.4 (67.0)	34.3 (102.9)	
Median (Q1-Q3)	8.3 (2.8-33.2)	5.2 (2.4-27.2)	p^MW^ = 0.013
Fiber [g/d]			
Mean (SD)	4.5 (1.6)	4.7 (2.0)	
Median (Q1-Q3)	4.2 (3.4-5.4)	4.2 (3.3-5.7)	p^MW^ = 0.981
Vitamin C [mg/d]			
Mean (SD)	93.1 (50.9)	99.4 (62.4)	
Median (Q1-Q3)	78.7 (57.9-117.6)	81.6 (55.2-129.5)	p^MW^ = 0.464
Calcium [g/d]			
Mean (SD)	0.72 (0.30)	0.79 (0.41)	
Median (Q1-Q3)	0.65 (0.50-0.86)	0.69 (0.50-0.93)	p^MW^ = 0.025
Dietary iron [mg/d]			
Mean (SD)	14.5 (4.9)	15.7 (6.7)	
Median (Q1-Q3)	13.6 (11.2-16.7)	14.0 (11.1-18.2)	p^MW^ = 0.036
Taking mineral supplements			df = 1
[n, (%)]	121 (14.2%)	105 (14.9%)	p^chi^ = 0.676

*MW* Mann–Whitney test, *df* degrees of freedom; *chi*-chi-square test.

The main aim of the study was to analyze the role of dietary calcium intake in the development of colorectal cancer. An increase of dietary calcium intake by 100 mg/day was associated with 5% decrease of colorectal cancer risk (OR = 0.95, 95% CI: 0.92-0.99), and this was significant also after adjustment for several covariates (Model 3 in the Table 3). Analyses performed for colon and rectal cancer separately, revealed that the effect of calcium was even greater for colon (OR = 0.93; 95% CI: 0.89-0.98) but was not observed for rectal cancer. Similar tendency was observed in the analyses investigating the effect of 1000 mg calcium/day increase. The cancer risk was significantly decreased by 37% for colorectal cancer, and by 50% for colon cancer, but was not significant for rectal cancer.

**Table 3:** Calcium intake and odds ratio for colorectal cancer, colon cancer and rectal cancer

**Dietary calcium**	**Model 1**			**Model 2**			**Model 3**		
	**OR**	**95% CI**	**P**	**OR**	**95% CI**	**P**	**OR**	**95% CI**	**P**
**Colorectal cancer**									
For 100 mg/day increase	0.95	0.92-0.98	<0.001	0.95	0.92-0.98	0.001	0.95	0.92-0.99	0.014
For 1000 mg/day increase	0.58	0.44-0.78	<0.001	0.61	0.45-0.82	0.001	0.63	0.44-0.91	0.014
<= 1000 mg/day	1 (ref.)			1 (ref.)			1 (ref.)		
>1000 mg/day	0.65	0.50-0.85	0.002	0.66	0.50-0.87	0.003	0.70	0.51-0.96	0.025
Dietary fiber <6 g/day									
For 100 mg/day increase	0.97	0.94-1.01	0.174	0.98	0.95-1.02	0.411	0.96	0.92-1.01	0.093
For 1000 mg/day increase	0.78	0.54-1.12	0.174	0.86	0.59-1.24	0.411	0.69	0.44-1.06	0.093
<= 1000 mg/day	1 (ref.)			1 (ref.)			1 (ref.)		
>1000 mg/day	0.85	0.60-1.23	0.409	0.91	0.63-1.32	0.628	0.87	0.59-1.30	0.508
Dietary fiber > =6 g/day									
For 100 mg/day increase	0.91	0.85-0.97	0.003	0.91	0.85-0.97	0.005	0.91	0.84-0.98	0.013
For 1000 mg/day increase	0.38	0.20-0.73	0.003	0.39	0.20-0.76	0.005	0.38	0.18-0.81	0.013
<= 1000 mg/day	1 (ref.)			1 (ref.)			1 (ref.)		
>1000 mg/day	0.54	0.33-0.87	0.011	0.54	0.33-0.88	0.013	0.48	0.27-0.85	0.012
**Colon cancer**									
For 100 mg/day increase	0.95	0.91-0.98	0.004	0.95	0.91-0.98	0.006	0.93	0.89-0.98	0.007
For 1000 mg/day increase	0.58	0.40-0.84	0.004	0.58	0.40-0.85	0.006	0.50	0.31-0.83	0.007
<= 1000 mg/day	1 (ref.)			1 (ref.)			1 (ref.)		
>1000 mg/day	0.58	0.40-0.83	0.003	0.59	0.41-0.85	0.005	0.54	0.35-0.83	0.005
Dietary fiber <6 g/day									
For 100 mg/day increase	0.98	0.93-1.02	0.273	0.98	0.94-1.03	0.405	0.94	0.89-0.998	0.042
For 1000 mg/day increase	0.78	0.50-1.22	0.273	0.82	0.52-1.30	0.405	0.55	0.31-0.98	0.042
<= 1000 mg/day	1 (ref)			1 (ref)			1 (ref)		
>1000 mg/day	0.76	0.47-1.22	0.248	0.82	0.50-1.34	0.428	0.72	0.43-1.23	0.232
Dietary fiber > =6 g/day									
For 100 mg/day increase	0.90	0.83-0.98	0.014	0.90	0.82-0.98	0.020	0.88	0.79-0.98	0.022
For 1000 mg/day increase	0.34	0.15-0.81	0.014	0.33	0.13-0.84	0.020	0.28	0.10-0.83	0.022
<= 1000 mg/day	1 (ref)			1 (ref)			1 (ref)		
>1000 mg/day	0.47	0.25-0.88	0.019	0.46	0.24-0.90	0.022	0.37	0.18-0.79	0.010
**Rectal cancer**									
For 100 mg/day increase	0.95	0.92-0.99	0.006	0.96	0.92-0.99	0.016	0.97	0.93-1.02	0.233
For 1000 mg/day increase	0.61	0.43-0.87	0.006	0.65	0.45-0.92	0.016	0.77	0.49-1.19	0.233
<= 1000 mg/day	1 (ref)			1 (ref)			1 (ref)		
>1000 mg/day	0.72	0.52-1.00	0.053	0.73	0.52-1.02	0.064	0.85	0.58-1.25	0.414
Dietary fiber <6 g/day									
For 100 mg/day increase	0.98	0.94-1.02	0.308	0.99	0.95-1.03	0.601	0.97	0.93-1.03	0.330
For 1000 mg/day increase	0.80	0.52-1.23	0.308	0.89	0.57-1.38	0.601	0.77	0.46-1.29	0.330
<= 1000 mg/day	1 (ref)			1 (ref)			1 (ref)		
>1000 mg/day	0.96	0.62-1.48	0.844	0.99	0.63-1.54	0.951	0.95	0.59-1.53	0.826
Dietary fiber > =6 g/day									
For 100 mg/day increase	0.92	0.85-0.996	0.039	0.92	0.85-1.00	0.051	0.91	0.83-1.01	0.066
For 1000 mg/day increase	0.43	0.19-0.96	0.039	0.45	0.20-1.004	0.051	0.41	0.16-1.06	0.066
<= 1000 mg/day	1 (ref)			1 (ref)			1 (ref)		
>1000 mg/day	0.61	0.33-1.11	0.107	0.62	0.34-1.15	0.128	0.57	0.27-1.20	0.138

Model 1 –univariable logistic regression.

Model 2 –adjusted for age, gender, education.

Model 3 –adjusted for covariates from Model 2 and fish consumption [servings/week], fruit, raw and cooked vegetable consumption [servings/day], fiber [g/day], vitamin C [mg/day], dietary iron [<=14,03 mg/day; >14,03 mg/day], alcohol consumption [g/week], adult lifetime recreational physical activity [METs-h/week], BMI [kg/m^2^], smoking [current, ex-smokers, non-smokers], taking mineral supplements [yes/no].

The effect of higher against lower level of dietary calcium was also analyzed. It was found that higher (>1000 mg/day) dietary calcium was associated with 30% (OR = 0.70, 95% CI: 0.51-0.96) decrease of colorectal cancer (in general) and with 46% (OR = 0.54; 95% CI: 0.35-0.83) decrease of colon cancer risk. Our investigation failed to show an association with rectal cancer (Table 3). Additional analyses considering the effect of smaller amounts of dietary calcium didn’t show the positive effect across categories of less than 1000 mg/day.

The second aim of our investigation was to verify the hypothesis that the protective effect of calcium depends on the level of fiber intake. The interaction between dietary calcium (>1000 mg/day; <=1000 mg/day) and fiber (continuous) was statistically significant considering all colorectal cancer cases (p for interaction in the full multivariable logistic regression model = 0.003) as well as colon cancer cases (p for interaction in the full model = 0.015). When rectal cancer cases were tested, p for interaction was insignificant (p for interaction in the full model = 0.062).

As a next step we investigated the effect of dietary calcium across different categories of fiber consumption. It was revealed that among individuals who consumed less fiber (<6 g/day) the effect of calcium was lower and statistically insignificant. Contrarily, those who consumed more fiber had stronger calcium associated decrease of colorectal and (when considered cancer site) colon cancer risk. This was not proven for rectal cancer (Table 3).

Finally, we analyzed the risk of colorectal, colon and rectal cancer across different levels (categories) of fiber intake and potential effect modification by the intake of dietary calcium. It was observed decreased risk with the increase of calcium intake at every level of fiber intake with the greatest risk reduction for the highest level of calcium and fiber intake (OR = 0.23). Similar pattern was observed for colon cancer, however estimates in the subgroup consuming the lowest amount of calcium (<1000 mg/day) were insignificant. Analysis performed for rectal cancer failed to show significant results (Table 4).

**Table 4:** Calcium-fiber interplay in the risk of colorectal cancer (CRC), colon cancer (CC) and rectal cancer (RC)

**Fiber**	**Calcium**	**<1000 mg/d**	**1000-2000 mg/d**	**>2000 mg/d**
<6 g/d		1 (ref.)	CRC: 0.55 (0.38-0.78)	CRC: 0.39 (0.22-0.71)
			CC: 0.47 (0.29-0.74)	CC: 0.28 (0.13-0.62)
			RC: 0.65 (0.41-1.01)	RC: 0.54 (0.26-1.12)
6-8 g/d		CRC: 0.58 (0.34-0.99)	CRC: 0.42 (0.24-0.73)	CRC: 0.30 (0.15-0.61)
		CC: 0.60 (0.31-1.19)	CC: 0.36 (0.18-0.74)	CC: 0.22 (0.09-0.55)
		RC: 0.60 (0.31-1.17)	RC: 0.50 (0.25-1.02)	RC: 0.42 (0.17-1.03)
>8 g/d		CRC: 0.44 (0.20-0.98)	CRC: 0.32 (0.14-0.71)	CRC: 0.23 (0.09-0.56)
		CC: 0.47 (0.17-1.30)	CC: 0.28 (0.10-0.78)	CC: 0.17 (0.05-0.54)
		RC: 0.47 (0.17-1.30)	RC: 0.39 (0.14-1.07)	RC: 0.32 (0.10-1.02)

Stratum specified odds ratios with 95% confidence intervals for multivariable logistic regression* which include interaction terms between the exposure (calcium intake) and effect modifier (fiber intake).

*- adjusted for age [years], gender, education, fish consumption [servings/week], fruit, raw and cooked vegetable consumption [servings/day], fiber [g/day], vitamin C [mg/day], dietary iron [<=14,03 mg/day; >14,03 mg/day], alcohol consumption [g/week], adult lifetime recreational physical activity [METs-h/week], BMI [kg/m^2^], smoking [current, ex-smokers, non-smokers], taking mineral supplements [yes/no].

## Discussion

Our study supports evidence showing the protective effect of calcium in the development of colorectal cancer. It has been investigated the effect of high doses (over 1000 mg/day) of dietary calcium. In general our findings suggest the presence of a relationship for colon cancer and failed for rectal cancer, what is consistent with some other published results [30,31]. Our study has proved the positive effect showing about 7% decrease in the risk of developing colon cancer for every 100 mg/day increase of dietary calcium, and more than 40% decrease among these who consumed more than 1000 mg/day as compared to lower consumption level. Moreover our investigation suggests the presence of interaction between fiber and calcium and consistent decrease of the risk of colorectal cancer with the increase of calcium and fiber intake. These results were strengthen for colon cancer, and the effect was not proven for rectal cancer.

In our study, we decided to investigate the role of relatively high amount of dietary calcium. Our decision was driven by the results of other studies showing the decrease of the CRC risk among individuals with the highest level of consumption (the ATBC study: >1789 mg/d RR = 0.6 [32]; the BCDDP study: >830 vs. <412 mg/day RR = 0.62 for colon but not for rectum) [33] and among patients taking supplements of 1200–2000 mg/d [34] or among these with high total calcium intake (>1270 vs. <472 mg/day, RR = 0.69 for colon) [33]. Some beneficial effect was also suggested by preliminary results of our study [35,36]. Thus, our results are consistent with these findings, and, similar to other studies, in our investigation the beneficial effect and an interaction was not observed for lower doses of dietary calcium. Besides, we have tried to verify the effect of vitamins, however our results were not consistent and partially insignificant [36].

An additional benefit of our study is the investigation of interaction effect between calcium and fiber. Both fiber and calcium intake have shown beneficial effect and considerable decrease of colon cancer risk. The benefit was not proven for rectal cancer. On the basis of the available data we were not able to distinguish if the lack of significance was a result of either the consequence of the sample size in relation to the smaller effect or the fact that there is no association for this cancer site. However, the benefit was observed when colon and rectal cancers were analyzed together.

The reason why dietary calcium may provide additional benefit when added to fiber is that there are different physiological functions of both dietary items. Protective effect of dietary fiber is explained by its role of bulking up the stool, there diluting carcinogens [37], by speeding up transit time of the bolus [38] and decreasing contact time between carcinogens and the large bowel [39]. Moreover, fiber has been postulated to bind to and to dilute (and thus to inactivate) carcinogens [40]. Additionally, fermentation of fiber leads to the production of short chain fatty acids which were observed to promote cell differentiation or induce apoptosis [41]. Dietary fiber is also closely associated with other potentially protective dietary items like antyoxidative vitamins and minerals because all of them are consumed as fruits and vegetables.

The role of calcium has also been investigated intensively so far. It has been observed that an intake of calcium increases the formation of insoluble calcium-phosphate-bile acid complexes in feces, decreases the concentration of soluble fatty acids and decreases lytic activity of fecal water [42]. Calcium is also suspected to promote differentiation and restraining growth of colonic cells through intracellular release of calcium, calmodulin activation and phosphorylation of intracellular enzymes [43].

The point requiring elucidation is how fiber and dietary calcium may additionally interplay and decrease the risk of colon cancer. One possible explanation is fiber’s role in the absorption of calcium, which increases with the decrease of pH [44]. Fiber as a source of short chain fatty acids decreases the pH of feces [45] and thus may increase absorption of calcium. Serum calcium, however, is strictly regulated by homeostatic regulation [46], and consequently, it is less likely that the beneficial effect of dietary calcium depends on the serum concentration. Nevertheless, it was found that calcium may act through the activation of calcium-sensing receptors present on the luminal surface of intestinal epithelial cells [47]. Their activation can influence cell differentiation [48]. Finally, all together, may explain the presence of modification effect between calcium and fiber.

There is limited number of published data showing an interaction between intake of dietary fiber and calcium and study results are inconsistent, as some early investigations provided insignificant results [49,50]. Later, the Wheat Bran Fiber (WBF) Study –an over 36 months of follow-up trial of cereal fiber supplements, showed, that an inverse association between total calcium intake and a risk of the occurrence of colorectal adenomas was stronger in the low fiber than in the high fiber group [51]. In the European Cancer Prevention Organization (ECP) Intervention Study -a randomized intervention trial of calcium and fiber supplementation, significant interaction between the level of baseline dietary calcium and fiber supplementation was observed in the fiber arm (however with increased risk of adenoma recurrence among these who consumed more dietary calcium). On the contrary, among those who consumed more dietary fiber at baseline and were in the calcium arm in the aforementioned study, a decreased (though insignificant) risk was observed [51]. The beneficial effect of both calcium and fiber was observed in the Calcium Polyp Prevention Study, as among individuals in the third tertile of consumption of dietary fiber the supplementation of 1200 mg/day of calcium showed 42% decrease of tubular adenoma risk when they were compared to the placebo group [52].

Our results are strengthened by our relatively large sample size, the ability to perform analyses in subgroups of colon and rectal cancer cases, a detailed assessment of dietary habits and a possibility to take into account several covariates. But, there are also some features requiring consideration. One is the role of vitamin D. Research has shown an inverse association between intake of vitamin D and CRC risk [53,54]. In our study we were not able to investigate the effect of calcium across different levels of vitamin D directly as the data about consumption of vitamin D was not available for us. Instead of this, we had [and adjusted for] information about the level of fish consumption, the main source of dietary vitamin D in our population (as taking supplements is very rare among adults, except treatment for osteoporosis, but such individuals were not included into the study). Additionally, logistic regression models were adjusted for leisure time physical activity being a surrogate of potential exposure to sunlight UVB—a determinant of 25(OH)D.

The presented study is not free from any limitations. One is the possible effect of supplementary calcium. According to the protocol, only information about general mineral supplementation was available. This was relatively rare, as only 14.5% took mineral supplements and sub-group analyses performed only among individuals who reported no mineral supplement use, didn’t influence final results. Another possible covariate we were not able to account for was taking NSAIDs (non-steroid anti-inflammatory drugs). Moreover, the retrospective design may be associated with bias—especially recall bias and selection bias. To minimize an effect of health conditions on the reported diet we asked about dietary habits 5 years before the presence of gastrointestinal symptoms (among cases) or before the diagnosis (among controls). Thus, we think that even if the effect of memory degradation and imperfect recall decreased the power of our study, our results shouldn’t be considerably skewed. We performed our study using hospital based controls. This may be another source of bias; however if dietary habits among controls were worse than among the general population of healthy adults, the observed benefit would be higher. Additionally, interviewers were not aware of the respondent’s diagnosis (they collect also this information) and could make stronger the association between calcium intake and colorectal cancer than it really is. This is one of the main concern in case–control studies. In our study however, interviewers were not informed of the purpose of the study or what kind of data will be investigated further. The dietary interview consisted of 148 questions, and thus it was less likely that interviewers consistently skewed (requires more details from controls) dietary data, especially regarding items containing calcium. All in all, we belief that some kind of differential bias might exist but its effect was small.

Several molecular studies to explain the role of calcium has been performed to date. Although these results haven’t been consistent either, it was observed that the effect of dietary calcium may depend on the genotype of vitamin D receptor (VDR) gene. Considering polymorphism within start codon of VDR (*FokI*), the risk of developing CRC was more than two-fold greater for individuals with ff genotype and low calcium intake. This relationship finally was alleviated by high intake of calcium [55]. Research on the *BsmI* and *poly-A* VDR polymorphism showed significant risk reduction for rectal cancer among individuals with SS or BB genotypes and accompanying low calcium intake. Contrary, SS or BB genotype individuals consuming high level of calcium had reduced risk of colon cancer [56]. The protective effect of high dietary calcium intake for these with BB genotype of the VDR *BsmI* has been also observed in some other investigations [57]. There was also a study on interactions between polymorphism of adenomatous polyposis coli gene (APC), dietary calcium and fiber intake and CRC showing that the group of high both calcium and fiber intakes was associated with about 50% lower risk of CRC among individuals with DV or VV genotype of APC [58]. On the whole, genetic polymorphisms might explain some differences also in our study, however this information was not available either.

In summary, our study confirmed the protective effect of higher doses of dietary calcium in the risk of colon cancer. This relationship was observed across different levels of dietary fiber, and the beneficial effect of dietary calcium depended on the level of dietary fiber suggesting modification effect of calcium and fiber. Further efforts are needed to prove this association, and also across higher levels of dietary fiber intake.

## Conclusions

1. The study confirmed beneficial effect of calcium consumption in the protection of colon cancer. The benefit was not confirmed for rectal cancer.

2. The protective effect was dependent on dosage, and was more clearly observed among individuals with dietary calcium over 1000 mg/day.

3. The study suggests that intake of calcium may be an effect modifier in the relationship between fiber consumption and the development of CRC.

4. The results support other evidence for the potential benefit of an increase of consumption of food reach in both calcium and fiber.

## Abbreviations

APC: Adenomatous polyposis coli; CC: Colon cancer; CRC: Colorectal cancer; RC: Rectal cancer; SFFQ: Semi-quantitative food frequency questionnaire; VDR: Vitamin D receptor.

## Competing interests

The authors declare that they have no competing interests.

## Authors’ contributions

AG participated in the design and coordination of the second part of the study, carried out data management, performed the statistical analyses, conceived, drafted and finalized the manuscript. EST and MA collected the data and helped to draft the manuscript. All authors read and approved the final manuscript.
